# The roots of COVID-19 vaccine hesitancy: evidence from Hungary

**DOI:** 10.1007/s10865-022-00314-5

**Published:** 2022-05-14

**Authors:** András Bíró-Nagy, Áron József Szászi

**Affiliations:** Centre for Social Sciences, Hungarian Academy of Sciences Centre of Excellence, Budapest, Hungary

**Keywords:** COVID-19, Vaccine hesitancy, Conspiracy theories, Logistic regression, Relationship violence, Hungary

## Abstract

This research explores the determinants of vaccine hesitancy during the third wave of the COVID-19 pandemic in Hungary. This article utilizes data from in-person public opinion research conducted in Hungary (March 2021, *N* = 1000). Government supporters, older people (60 +) and COVID-19 survivors were more likely to accept vaccination, but these variables lose significance, once controlling for personal fears and pandemic-related attitudes. COVID-19 related fears and precautious behavior reduce, while general level of fears increase the probability of vaccine hesitancy. Fear from partner’s aggression and higher levels of financial security negatively correlate with vaccine hesitancy. Our study separately analyzes the effect of various pandemic-related conspiratorial beliefs on vaccine hesitancy. All analyzed false beliefs have a significant positive effect on vaccine hesitancy, but the strongest predictors are vaccine-related conspiracy theories (“microchip” and “population control” theories) and virus denial.

## Introduction

Vaccine hesitancy has become one of the major obstacles to vaccine acceptance, leading to poor health outcomes and mortality associated with the COVID-19 pandemic. Paradoxically, once COVID-19 vaccines became available, the level of vaccine refusal increased in some countries, and an estimated one fifth of people do not intend to receive the vaccine as a meta-analysis of 28 studies from 13 countries revealed (Robinson et al., 2021). Extensive studies on this subject are relatively recent and our research adds to this growing body of literature by providing observational evidence about vaccine hesitancy from Hungary.

The Hungarian case is especially intriguing, since the country ranked fourth globally regarding the total COVID-19-related deaths/capita on 8 February 2022 (Johns Hopkins University, 2022), while in the early stages of COVID-19 vaccination programmes it was one of the fastest EU-member regarding the pace of vaccination because of the early import of Russian Sputnik-V and Chinese Sinopharm vaccines (besides Pfizer, AstraZeneca and Moderna vaccines). Partly because of the mistrust in these ‘Eastern’ vaccines, and the Hungarian government’s close partnership with China and Russia, vaccination became the topic of heated political debates (Hungary Today, 2021). However, the politicization of vaccination is not exclusive to Hungary. A longitudinal study conducted in the US revealed that the supporters of the two major parties were polarized with regards to vaccine hesitancy (Republicans being hesitant to a larger extent), whereby the initial gap between the two groups widened in 2020, which could be corroborated to the power of political communication on them in their respective media echo chambers (Fridman et al., 2021).

Attention, scrutiny, and stakes are clearly much higher during this public health crisis than ever before in recent decades and therefore, there are several factors of vaccine hesitancy that have become more prominent in general. For example, new fronts of vaccine hesitancy were introduced into the mainstream as a survey conducted in Israel found that determinants of trust levels towards Covid-19 vaccines have increasing degrees of importance within geographical and technological dimensions, whereby whether the vaccine is coming from China, the US, or Russia and whether it employs mRNA, Vector, or traditional technologies also plays a role (Dror et al., 2021).

As a meta-analysis of fifteen studies revealed, general lack of trust is amongst the most listed reasons for being hesitant towards a potential Covid-19 vaccine, which could be presented – among others – in the forms of concerns towards the safety, the potential dangers of fast development, and misbelief in the communication that the disease is as severe as the science shows it to be (Troiano & Nardi, 2021). In addition to this, an ongoing working paper of the IMF found that the two most decisive motivating factors that reduce people’s intent to be vaccinated are firstly, mistrust in the ability of the government to provide an efficient vaccine and concerns about the potential side effects of taking the shot, which, if it receives widespread attention can result in a drop of vaccine demand by almost a third, further decreasing trust in Covid-19 vaccines (Dabla-Norris et al., 2021). A survey study on the 2018 defective vaccine scandal in China has brought forward evidence that ‘perceived negative publicity’ of vaccine qualities is capable of acting as a substantial predictor of one’s trust levels in the form of risk perception and information forwarding (Yan et al., 2021).

This research is exploratory regarding its goal aiming to identify the major determinants of vaccine hesitancy on the individual level by conducting a multi-variate analysis involving political, socio-economic and various cognitive, emotional and behavioral variables. Conspiratorial beliefs turned out to be major predictors of COVID-19 vaccine hesitance in several recently published studies (Murphy et al. 2021; Nazlı et al. 2022). Hence our study pays special attention to the role of pandemic-related conspiracy theories. Our goal was to find out what kind of COVID-19 and vaccine-related false beliefs are the best predictors of vaccine hesitancy.

## Background: public health communication, vaccine procurement and vaccine hesitancy in Hungary

All major stakeholders, such as the government, health care experts, and opposition politicians began the pursuit of large campaigns to popularize immunization against Covid-19 through vaccination in Hungary. The ‘Vaccination saves lives’ campaign of the Hungarian Government, whereby popular figures have promoted vaccination began in mid-April 2021 (Dömötör, 2021). At the beginning of May 2021, once vaccines became widely available, the government launched another major awareness campaign to tackle vaccine hesitancy financed by approximately 45 million euros (Magyar Közlöny, 2021). The opposition-led municipality of Budapest launched its own vaccine-promotion campaign also involving celebrities in February 2021 (Vaski, 2021a). Opposition parties and politicians also started to promote vaccination, for example with a joint video of the members of the opposition alliance, and with party statements promoting the need for vaccination to tackle the crisis (Hungary Today, 2021). A narrower campaign began in May 2021 by NGOs, National Minority Municipalities and journalists that aimed to popularize vaccines to the Roma community and help them to register for vaccination (Jász, 2021).

**Fig. 1:**
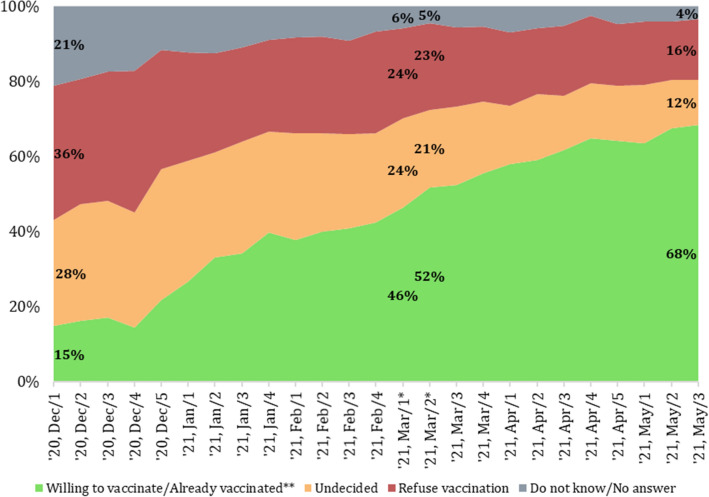
Change of attitudes toward COVID-19 vaccination between December 2020 and May 2021. Source: Hungarian Central Statistical Office [HSCO], 2021. *Note*: *The marked weeks overlap with the collection of the data analyzed in this article. **Those who were vaccinated with one dose or more against COVID-19

**Fig. 2:**
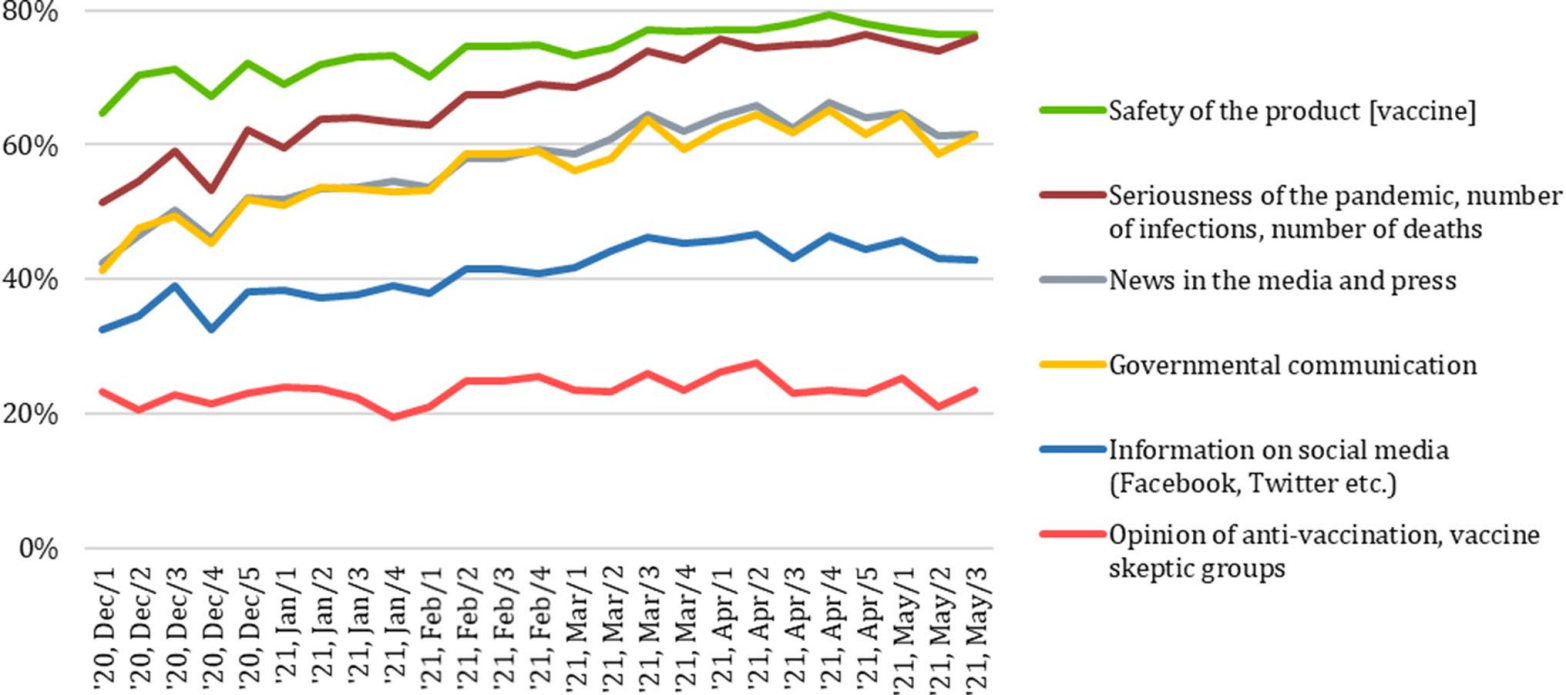
Factors influencing vaccine-related attitudes (December 2020–May 2021). Source: Hungarian Central Statistical Office [HSCO], 2021. *Note*: Based on survey respondents’ self-report, proportion of those who answered that a given factor influenced them “to a medium extent”, “to a large extent” or “completely”

It is reasonable to assume that vaccination promotion had a positive effect as willingness to take vaccination increased from 15 to 68% between December 2020 and May 2021 (see Fig. 1). However, several other potential factors could have led to the increase of vaccine confidence. A natural spiral of increasing vaccine confidence could occur, as more and more people getting vaccinated without experiencing any serious side-effects could have convinced hesitant people about the safety of vaccines. Personal relationships often have a significant influence on individual health practices (Rothman et al., 2020), hence many may have experienced social pressure to get vaccinated. There was also an increase in the share of those who claimed that governmental communication, news in the traditional and social media influenced their attitudes toward vaccination (see Fig. 2). Perceived safety of the vaccines and seriousness of the pandemic had more widespread impact on vaccination attitudes according to respondents’ self-report. Figure 2 also shows that the latter factor’s role sharply increased during the spring of 2021, the time of the third (and most lethal) wave of the COVID-19 pandemic in Hungary.

A Hungarian think-tank, Political Capital’s reports provide the most comprehensive picture about the evolution and present state of the novel anti-vaxxer movement in Hungary (Győri, 2021; Istrate, 2021). In March 2020, “fringe health experts who usually had a medical degree but worked as ‘alternative medicine’ service providers or entrepreneurs started to control fringe news threads, initially in a balanced, cautious manner” (Győri, 2021). In May 2020, however, the movement radicalized in its messaging and started to organize itself in an orderly manner. The self-declared experts launched a fringe news site called ‘Médiaforrás’ (‘Media Source’), established an organization called ‘Orvosok a Tisztánlátásért’ (‘Doctors for Clarity’) and organized a conference inviting conspiracy theorists from Russia and Germany. The movement radicalized even further during the second wave of the pandemic in Hungary (Autumn 2020): protests were organized and the narrative changed from virus-skepticism to conspiracy theories about the newly developed COVID-19 vaccines. Although the Hungarian Chamber of Doctors called for denouncing ‘Doctors for Clarity” in August 2020, the government did not rebuke the organization until 30 October (Istrate, 2021). Every fourth/fifth Hungarians took into consideration anti-vaxxer groups opinion when they decided about vaccination (see Fig. 2) proving that these voices effectively penetrated the public sphere in Hungary. The mainstream media provided a platform to the members of ‘Doctors for Clarity’ after the first wave of the pandemic, though vaccine-sceptic sources in Hungary are mainly Facebook pages and groups and fringe media sites; amongst these are several pro-Russian sites in Hungary (Győri, 2021; Istrate, 2021).

Political context further increases the complexity of vaccine hesitancy in Hungary. Besides the vaccines authorized by the European Medicines Agency (EMA), the Hungarian Government decided to obtain 5 million doses of Sinopharm and 2 million doses of Sputnik-V vaccines – (enough for roughly 25% and 10% of the population of Hungary, respectively) – within their vaccine procurement (Reuters, 2021). This action has facilitated a political debate whereby, on the opposition side, politicians argued that the Prime Minister is placing pressure on the Hungarian authorities to approve these vaccines under emergency use, meanwhile one of the opposition parties (Democratic Coalition) launched a petition against the use of Sinopharm as long as it does not gain approval from the European Medicines Agency (Vaski, 2021b). The politicization of this issue also came forward on the Government side, as Prime Minister Viktor Orbán declared at the end of January 2021 that he trusts the Chinese the most with developing the vaccine (Nagy, 2021) and confirmed that he had personally received Sinopharm (Euronews, 2021). The governing parties and the opposition accused one another of eroding trust in Eastern/Western vaccines and being anti-vaxxers (Hungary Today, 2021; Istrate, 2021). Meanwhile, the anti-vaxxer movement established a new party called ‘Normális Élet Pártja’ (‘Party for Normal Life’) in May 2021 (Győri, 2021), and the far-right party ‘Our Homeland’ took a clearly anti-lockdown and anti-vaxxer stance (Istrate, 2021).

### Data

The analyzed data originates from an in-person survey recorded by a public opinion research company, Závecz Research in Hungary between 2 and 11 March 2021. The questionnaire, written by the authors, was embedded in a longer, omnibus survey besides other questionnaires unknown to us. Our questionnaire is available amongst the Online Supplementary Materials. Besides our questions, Závecz Research provided us the answers to basic demographic questions and questions about the participants’ political preferences. Závecz Research informed us that the participants’ answers were anonymized, all personal data were deleted after the end of the project. Závecz Research handled the collection of respondents’ consents to participate in the public opinion research. The Research Ethics Committee of the Centre for Social Sciences (Budapest, Hungary) declared that the authors assessed the potential risks of the research and the planned management of the potential risks is sufficient from research ethics’ perspective.

1000 respondents’ answers were recorded, as it is a standard sample size in Hungary. Random, multi-stage, stratified technique was used for sampling to guarantee the representativity of the data, and survey weights were constructed to eliminate differences between the sample and population regarding basic demographic characteristics. Our data represents the adult population of the country regarding gender, age, education and residence.

## Methods

### Rationale for variable selection

Several studies analyzing the roots of vaccine hesitancy study basic demographic variables in some form (Freeman et al., 2020; Dable-Norris et al., 2021; Gerretsen et al., 2021, Murphy et al., 2021), hence we decided to include them as a starting point of our analysis. Besides the examples of other studies (Fridman et al., 2021; Ward et al., 2020), the above-described politicization of vaccination in Hungary gave us rationale to introduce political preferences to our variables as well. We also decided to look at variables within economic status, religious, and class identity, which are studied to an extent in the above-mentioned papers, and also in the likes of Endrich et al. (2009) that delved into analyzing the socioeconomic factors behind influenza vaccination uptake.

Recently published studies highlight the importance of revealing the psychological roots of COVID-19 vaccine hesitancy (Barello et al., 2021; Gerretsen et al., 2021; Murphy et al., 2021; Nazlı et al., 2022). Our study involved the general level of fears, as we assumed that vaccine hesitancy has affective roots. Evidence suggest that paranoia is indeed negatively associated with COVID-19 vaccine uptake (Murphy et al., 2021). Our research also analyzed the effects of social isolation and fear from relationship violence during quarantine. We consider these variables to be good proxies of the adverse psychological effects of the lockdown on the individual, which has been shown to have negative effect on vaccine hesitancy in the study of Gerretsen et al. (2021). Furthermore, social isolation might be negatively associated with a sense of collective responsibility, a predictor of COVID-19 vaccine confidence in the study of Barello et al. (2021).

Our survey included several questions measuring pandemic-related experiences, behaviors, emotions and conspiratorial beliefs. Including these variables enabled us to explore which COVID-19-related attitudes are the best predictors of vaccine hesitancy. We aimed to find out which conspiracy theories play the biggest role in the spread of vaccine hesitancy, therefore we measured beliefs in five different, pandemic-related conspiracy theories. We included two types of vaccine-related misinformation in our analysis: the “vaccination is population control” and “microchip in the vaccine” theories. Early research suggests that a major source of COVID-19 vaccine hesitancy amongst people of reproductive-age is the fear that inoculation causes infertility (Hsu et al., 2022). After the Emergency Use Authorization of the new vaccines in the US, the number of searches about COVID-19 vaccines and infertility sharply rose (Diaz et al., 2021). 13% of the unvaccinated US citizens have heard about the “vaccination causes infertility” theory according to a survey research carried out in January 2021 (Hamel et al., 2021). Rumors about vaccine-associated infertility are not new phenomena: similar unfounded beliefs were common in relation to parental hesitancy towards vaccinating their children against Human Papillomavirus (Schuler et al., 2013). “Microchip in the vaccine” theory had also been widespread before the COVID-19 pandemic, for instance in relation to H1N1 vaccines (Krekó et al., 2015). Novel versions of the theory state that the microchip (planted into people immunized against COVID-19) is activated with 5G technology (Önnerfors, 2021). Although no previous research analyzed the linkage between virus denial and vaccine hesitancy to our knowledge, we assumed that it would be a strong predictor of vaccine hesitancy. Our research also included two theories explaining the origin of the virus: one stating that China intentionally infected the world, another suggesting that pharmaceutical companies developed the virus. These theories were globally known as a result of politicians openly accusing China’s role in the pandemic and because of a viral quasi documentary about “big pharma conspiracy” (Önnerfors, 2021).

### Outcome variable

The outcome variable of our study is vaccine hesitancy, which we defined as being unwilling to get vaccinated or having doubts about getting vaccinated. A widely accepted definition of vaccine hesitancy is that the notion “refers to delay in acceptance or refusal of vaccines despite availability of vaccination services” (European Centre for Disease Prevention and Control, 2022a). Our conceptualization of vaccine hesitancy is slightly different because we work with survey data measuring attitudes instead of behaviors. We measured intent to get vaccinated because the data was recorded in the early phase of the vaccination campaign against COVID-19 in Hungary. Our vaccine hesitancy variable is conceptualized as a dichotomous variable based on the following survey questions: *“(1) Would you accept one of the vaccines against coronavirus?; Would you accept vaccination against coronavirus, if only… (2) …European/American-developed COVID-19 vaccines were available?; (3) …Russian-developed COVID-19 vaccines were available?; (4) …Chinese-developed COVID-19 vaccines were available?”* It is coded 1 if respondents answered either “Probably wouldn’t get vaccinated”, or “Definitely wouldn’t get vaccinated” or “Don’t know” to all intent to vaccinate questions. It is coded 0 otherwise, meaning that respondents are willing to accept at least one type of vaccine.

### Predictor variables

Since the main opposition parties in Hungary will run together in the next general elections, political preference is a categorical variable taking three values. It is coded "Government supporter" if a respondent would vote for "the list of Fidesz-KDNP", "Opposition supporter" if the answer is "the united opposition's list" and "Undecided voter" if the respondent refuses to answer or answer that he/she does not know. Relationship status categories are based on whether the respondent lives in a common household with a partner (in a relationship) or not (single); and whether he/she raises at least one child who is under 18 (with children) or not (without children). Fear from partner is coded 1 if a respondent answered that he/she is rather worrying or seriously worrying about “*Your partner becomes nervous, aggressive during home confinement*”, or already experienced it. We included a dynamic and a static variable for perceived economic status. The dynamic variable measures whether a respondent considers his/her financial situation to improve, worsen or be the same since the eruption of the pandemic. The static measure is based on a question asking respondents to choose the best description of their financial status. We constructed four indices used as independent variables, namely *Personal fear, COVID-19 fear, Precautious behavior* and *Social isolation indices.* These variables are the means of the answers to their composite questions (listed in Table A1 in the Online Appendix) inverted to a 0–1 scale.

Various conspiratorial beliefs were measured by asking respondents to evaluate the following statements on a 10-points scale (1 – Completely disagree, 10 – Completely agree). Microchip theory: *“With the COVID-19 vaccination, a microchip may be built in the body secretly”*; Population control theory: *“COVID-19 vaccination may cause infertility; the secret goal of the vaccination is population control.”*; No virus theory: *“Coronavirus does not exist; it is made up”*; China theory: *“Coronavirus was spread to the world by China in order to take the role of the world’s leading power”*; Pharma theory: *“Coronavirus was developed by pharmaceutical companies in order to help them sell their drugs and vaccines more easily”*. It was operationalized different ways in two different set of models (see Table 5, first as a continuous variable (answers inverted to 0–1 scale), second as a categorical variable (Non-believers: 1–3 answers, Hesitant: 4–7, Believers: 8–10). Further independent variables are based on answers to single survey questions. For details about survey questions and coding, see Table A2 in the Online Appendix.

### Data analysis

Logistic regressions were run to detect the major correlates of vaccine hesitancy. Survey weights were used in all models to increase the external validity of our results. Average marginal effects (showing change of probability of outcome variables) are displayed in the in-text tables to help interpret the results. Logistic regression coefficients (change of log-odds ratios) are reported in the Online Appendix. All statistical analyses were carried out using Stata 16 software, while the descriptive figures were made with R Studio.

In the first series of analyses (Table 4), political and demographic variables are included in the first model, additional independent variables are gradually involved in further models. Various COVID-19-related conspiratorial beliefs’ effects on vaccine hesitancy are analyzed in a separate set of models applying two different model specifications (Table 5). Conspiratorial beliefs’ effect on vaccine hesitancy is analyzed in separate models. In these analyses, we only control for political, demographic variables and COVID-19 experience to avoid multicollinearity problems. Conspiratorial beliefs are operationalized as continuous variables (0–1 scale measure) in the first set of models (Model 7-Model 11), and they are operationalized as categorical variables in the second set of models (Model 12-Model 16). The latter enables the separate analysis of believers and deniers of the different theories, people who are hesitant and those who are not willing to express their views about these theories.

## Results

### Descriptive statistics

Table 1 shows the distribution of the dependent variable of our study and the distribution of the answers to its component questions. Based on our multi-item measurement, three quarters (76%) of the adult Hungarians were vaccine confident, while one quarter (26%) of Hungarians were vaccine hesitant in March 2021. If we were to calculate vaccine hesitancy based on only one of the component questions, the ratio of vaccine confidents would be 8–25% points (pp) lower. Two thirds (68%) answered that they would probably or definitely get vaccinated or they were already vaccinated with “one of the vaccines” and with “the European/American vaccines”. The acceptance ratios of Russian and Chinese vaccines were much lower (52%).

**Table 1:** Distribution of vaccine confident/vaccine hesitant respondents

Overall attitudes toward vaccination						
	Vaccine confident people			Vaccine hesitant people		
	74%			26%		
General/origin-specific attitudes toward vaccines						
	Already vaccinated (%)	Definitely would get vaccinated (%)	Probably would get vaccinated (%)	Probably wouldn't get vaccinated (%)	Definitely wouldn't get vaccinated (%)	Do not know / No answer (%)
*Willingness to accept…*						
…One of the vaccines	6	30	32	14	13	5
…European/American vaccines	3	32	33	12	13	6
…Chinese vaccines	2	20	29	20	21	8
…Russian vaccines	2	20	29	20	19	9

The proportions are weighted

**Fig. 3:**
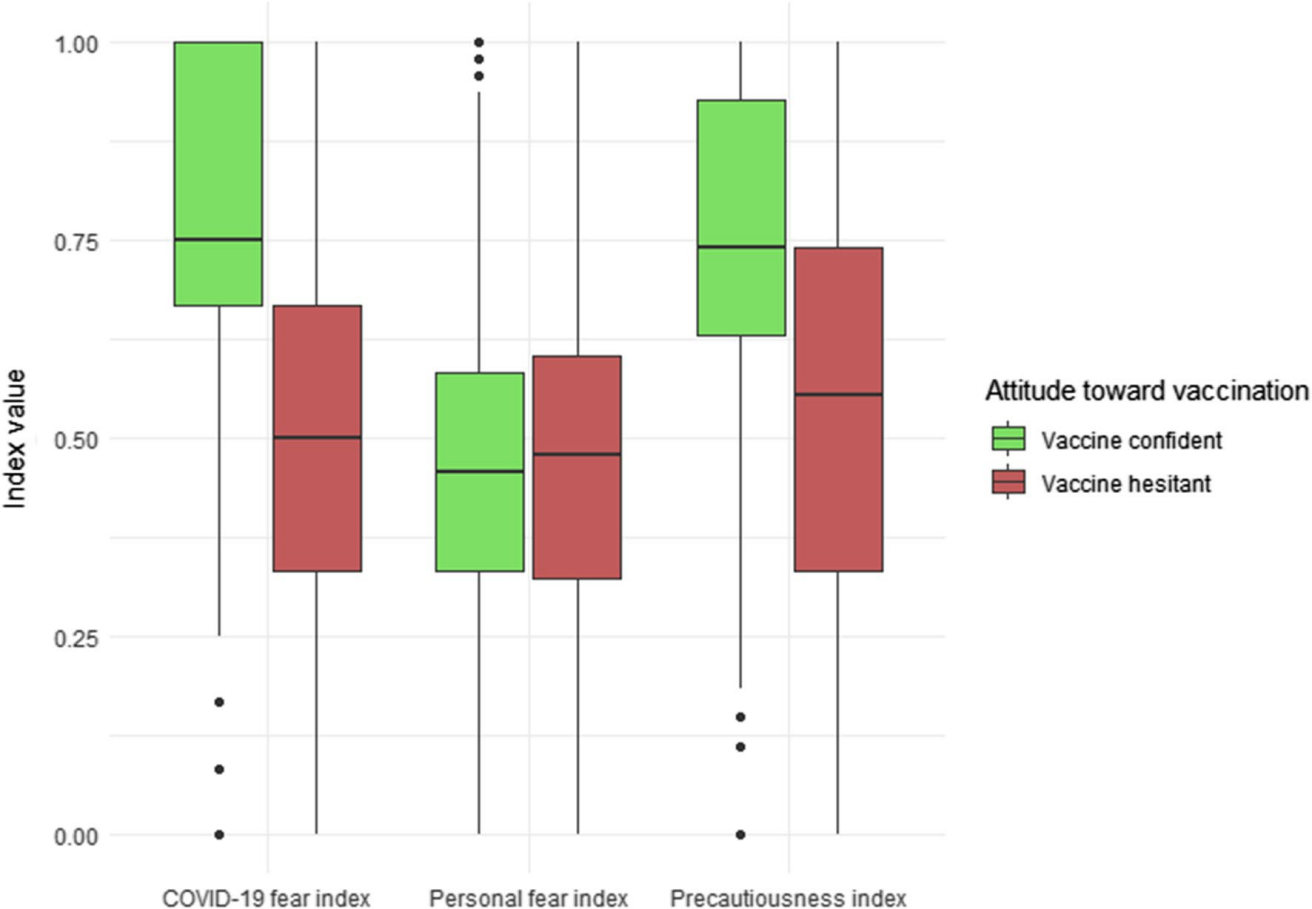
Distribution of COVID-19 and fear-related indices by attitudes toward vaccination

The distribution of COVID-19 and fear-related indices is presented in the box plots of Fig. 3. It is visible that COVID-19 fear index and Precautiousness index are significantly higher among the vaccine confident group than the vaccine hesitant group of our sample (two-tailed *t*-tests, *p* < 0.001). However, the personal fear index is not significantly different in the two groups.

The distribution of the categorical independent variables of our analysis are presented in Table 2. We separately presented the categories’ ratios within the total sample, vaccine confident and vaccine hesitant subgroups. We ran Pearson chi-squared tests for independence to see whether the two subgroups differ significantly regarding the given variables. There were significant difference in relation to the following variables: political preference (Chi2 = 36.9, *p* = 0.000), age groups (Chi2 = 39.6, *p* = 0.000), gender (Chi2 = 6.4, *p* = 0.011), family status (Chi2 = 17.1, *p* = 0.001), fear from partner (Chi2 = 5.6, *p* = 0.018), static financial status (Chi2 = 23.6, *p* = 0.000) and dynamic financial status (Chi2 = 17.1, *p* = 0.000), religiousness (Chi2 = 18.5, *p* = 0.000) and class identification (Chi2 = 13.3, *p* = 0.01).

**Table 2:** Distribution of categorical independent variables within the total sample, and vaccine confident/vaccine hesitant groups

	Total sample	Vaccine confident people	Vaccine hesitant people
*Political preferences*			
Government voter	37%	41%	23%
Opposition voter	38%	38%	40%
Undecided voter	25%	21%	37%
*n*	973	730	243
*Education*			
Elementary or less	29%	30%	26%
Vocational school	22%	21%	26%
Secondary school	31%	31%	33%
Higher education	18%	19%	15%
*n*	1000	744	256
*Age group*			
18–29	18%	15%	26%
30–39	20%	18%	24%
40–49	17%	16%	19%
50–59	17%	18%	16%
Older than 60	28%	33%	15%
n	995	740	255
*Settlement type*			
Budapest	18%	19%	16%
County seat	17%	16%	19%
Town	35%	36%	35%
Village	29%	29%	29%
*n*	1000	744	256
Gender			
Male	47%	44%	54%
Female	53%	56%	46%
*n*	1000	744	256
*COVID-19 experience*			
Did not get infected	97%	96%	98%
COVID-19 survivor	3%	4%	2%
n	1000	744	256
*Family status*			
Single, no child	29%	28%	31%
In a relationship, no child	42%	45%	33%
Single parent	3%	3%	2%
In a relationship, with child	27%	24%	34%
*n*	998	742	256
*Fear from partner*			
No fear from partner	74,4	72,3	80,3
Have fear from partner	25,6	27,7	19,7
*n*	1000	744	256
*Financial status (static)*			
Living on loans/aid	4%	4%	5%
Live off savings	9%	8%	11%
income barely covers living	54%	51%	62%
No financial problem, but cannot save up	21%	23%	16%
Can save up a little	11%	13%	5%
Can save up significant amount	1%	2%	0%
*n*	953	714	239
*Financial status (dynamic)*			
Fin. sit. worsened	39%	35%	49%
Fin. sit. did not change	59%	62%	51%
Fin. sit. improved	2%	2%	0%
n	999	744	255
*Religiousness*			
Not religious	48%	44%	59%
Religious	52%	56%	41%
*n*	1000	744	256
*Class identification*			
Lower class	20%	19%	24%
Lower middle class	39%	37%	45%
Middle class	37%	40%	27%
Upper middle class	3%	3%	4%
Upper class	0%	0%	0%
*n*	971	725	246

The proportions are weighted values, while the frequencies (n) are not weighted in the table above

Because our research applies two operationalizations of conspiratorial beliefs, we separately present these results. Table 3 shows the distribution of the level of beliefs in conspiracy theories, as a categorical variable. “China theory” is the most accepted: more than half of our respondents were uncertain or tend to believe this theory. The answers broke down along similar lines when it came to the “Pharma theory”. The other theories are rejected to a much larger extent, although the ratio of those who could not or did not want to answer the “Population control theory” was quite high compared to the other theories.

**Table 3:** Distribution of pandemic-related conspiratorial beliefs in the total sample and by attitudes toward vaccination (categorical variable)

	Do not believe (1–3)	Uncertain (4–7)	Believe (8–10)	Do not know / Refuse to answer
	Total sample			
China intentionally infected the world	35%	35%	17%	13%
Pharma companies created the virus	39%	35%	14%	12%
Vaccination is population control	46%	23%	6%	26%
Microchip in the vaccines	66%	17%	5%	13%
Virus denial	65%	18%	5%	12%
	Vaccine confident people			
China intentionally infected the world	39%	36%	14%	11%
Pharma companies created the virus	44%	35%	12%	9%
Vaccination is population control	52%	19%	4%	25%
Microchip in the vaccines	72%	15%	3%	10%
Virus denial	73%	14%	3%	9%
	Vaccine hesitant people			
China intentionally infected the world	23%	33%	27%	17%
Pharma companies created the virus	25%	35%	21%	19%
Vaccination is population control	28%	33%	10%	29%
Microchip in the vaccines	49%	20%	11%	20%
Virus denial	42%	29%	11%	19%

The proportions are weighted

**Fig. 4:**
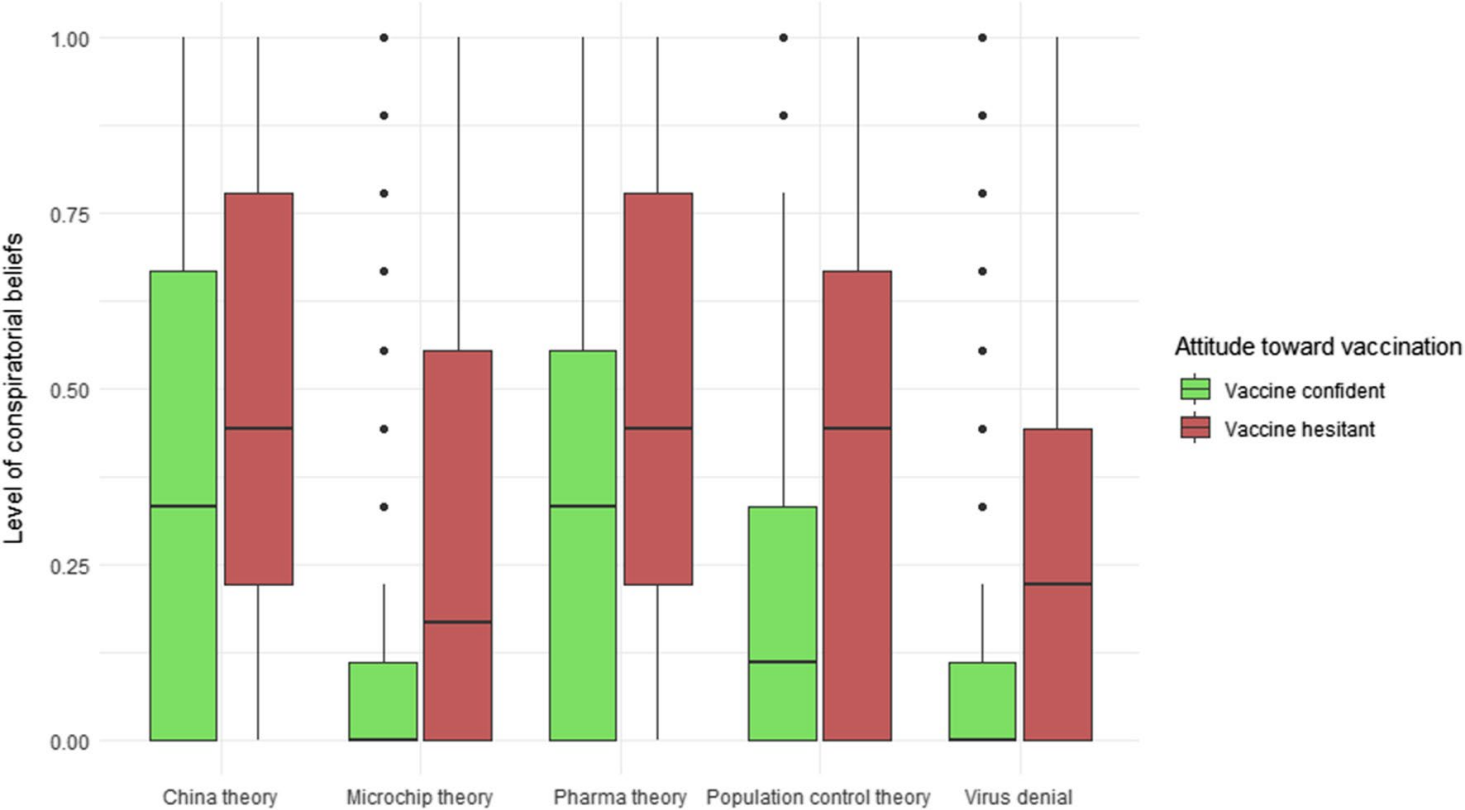
Distribution of pandemic-related conspiratorial beliefs by attitudes towards vaccination (continuous variable)

The distribution of conspiratorial beliefs, as a continuous variable is visualized in Fig. 4. The level of these beliefs is higher amongst vaccine hesitant people in relation to all examined theories (two-tailed *t*-tests, *p* < 0.001).

### Statistical analyses

Table 4 explores the political, demographic, social, economic roots of vaccine hesitancy. Political preference, age and gender were significant in Model 1. The probability of being vaccine hesitant was 10% points higher amongst opposition voters (*p* < 0.001), and 19 pp higher amongst undecided voters (*p* < 0.001) compared to government supporters. People older than 60 were 22 pp less likely (*p* < 0.001), people aged 50–59 were 11 pp less likely (*p* < 0.05) to be hesitant compared to adults under 30. Women were 6 pp less likely to be hesitant than men (*p* < 0.05).

**Table 4:** Political, demographic, social, economic roots of vaccine hesitancy, weighted logistic regression models

	Model 1		Model 2		Model 3		Model 4		Model 5		Model 6	
VARIABLES	AME	95% CI	AME	95% CI	AME	95% CI	AME	95% CI	AME	95% CI	AME	95% CI
*Political preferences*												
Government voter	Reference		Reference		Reference		Reference		Reference		Reference	
Opposition voter	.10***	[.04,.16]	.10***	[.04,.16]	.03	[−.03,.09]	.02	[−.04,.08]	.01	[−.05,.08]	.00	[−.06,.07]
Undecided voter	.19***	[.12,.26]	.20***	[.13,.27]	.11**	[.04,.18]	.11**	[.03,.18]	.09*	[.01,.16]	.08*	[.00,.15]
*Demographic variables*												
Elementary school or less	Reference		Reference		Reference		Reference		Reference		Reference	
Vocational school	−.02	[−.10,.06]	−.02	[−.10,.06]	−.01	[−.09,.06]	−.02	[−.09,.06]	.01	[−.06,.08]	−.00	[−.08,.07]
Secondary school	−.03	[−.10,.05]	−.03	[−.11,.05]	.01	[−.06,.09]	.02	[−.05,.10]	.05	[−.02,.13]	.05	[−.04,.14]
Higher education	−.07	[−.15,.01]	−.07	[−.15,.01]	−.06	[−.13,.02]	−.04	[−.12,.03]	.01	[−.08,.09]	−.00	[−.11,.10]
18–29	Reference		Reference		Reference		Reference		Reference		Reference	
30–39	−.04	[−.14,.05]	−.04	[−.14,.05]	.01	[−.08,.10]	.02	[−.08,.12]	.01	[−.08,.11]	.01	[−.09,.10]
40–49	−.06	[−.16,.04]	−.06	[−.15,.04]	−.01	[−.10,.08]	−.01	[−.10,.09]	.00	[−.09,.10]	.00	[−.10,.10]
50–59	−.11*	[−.21,−.02]	−.11*	[−.21,−.01]	−.03	[−.12,.05]	−.03	[−.13,.06]	−.03	[−.12,.06]	−.03	[−.12,.06]
60 or older	−.22***	[−.31,−.14]	−.22***	[−.31,−.14]	−.10*	[−.19,−.02]	−.10*	[−.19,−.02]	−.10*	[−.19,−.01]	−.10*	[−.19,−.01]
Budapest	Reference		Reference		Reference		Reference		Reference		Reference	
County seats	.07	[−.02,.17]	.07	[−.02,.17]	.03	[−.06,.12]	.03	[−.06,.12]	.04	[−.05,.13]	.04	[−.05,.14]
Towns	.00	[−.07,.08]	.01	[−.07,.09]	−.00	[−.08,.08]	.00	[−.08,.09]	.02	[−.06,.10]	.02	[−.06,.11]
Villages	.01	[−.07,.09]	.01	[−.07,.09]	.04	[−.04,.12]	.03	[−.06,.11]	.04	[−.05,.12]	.04	[−.05,.12]
Male	Reference		Reference		Reference		Reference		Reference		Reference	
Female	−.06*	[−.11,−.00]	−.06*	[−.11,−.00]	−.04	[−.09,.01]	−.04	[−.09,.01]	−.04	[−.09,.01]	−.04	[−.10,.01]
*COVID−19 experience*												
COVID-19 survivor			−.13*	[−.24,−.01]	−.06	[−.20,.08]	−.07	[−.20,.05]	−.05	[−.18,.08]	−.04	[−.17,.09]
*Fear and precaution indices*												
Personal fear index (0−1)					.12	[−.00,.25]	.17*	[.04,.31]	.15*	[.02,.29]	.15*	[.01,.29]
Covid-19 fear index (0−1)					−.51***	[−.61,−.40]	−.51***	[−.61,−.41]	−.50***	[−.61,−.40]	−.51***	[−.61,−.41]
Precautious behavior index (0−1)					−.17**	[−.30,−.04]	−.19**	[−.32,−.06]	−.19**	[−.32,−.06]	−.18**	[−.31,−.04]

*Personal relationships*												
Single. no children							Reference		Reference		Reference	
In a relationship. no children							.06	[−.01,.13]	.07	[−.00,.15]	.08*	[.00,.15]
Single parent							−.09	[−.21,.02]	−.10	[−.21,.00]	−.10	[−.20,.01]
In a relationship. with children							.04	[−.04,.12]	.05	[−.03,.13]	.05	[−.03,.13]
Social isolation index (0−1)							.13	[−.06,.33]	.08	[−.11,.27]	.08	[−.12,.27]
Fear from partner							−.09**	[−.15,−.03]	−.10**	[−.16,−.04]	−.11***	[−.16,−.05]
*Economic status*												
Fin. situation didn't change									Reference		Reference	
Fin. situation worsened									.06*	[.00,.11]	.06*	[.01,.12]
Fin. situation improved									−.15**	[−.25,−.06]	−.15***	[−.24,−.07]
Living on loans/aid									−.08	[−.19,.02]	−.07	[−.18,.04]
Live off savings									−.04	[−.12,.05]	−.04	[−.12,.05]
Income barely covers living									Reference		Reference	
No financial problem. but cannot save up									−.08*	[−.15,−.01]	−.08*	[−.15,−.01]
Can save up a little									−.13**	[−.22,−.05]	−.13**	[−.22,−.04]
Can save up significant amount									−.19***	[−.29,−.09]	−.21***	[−.30,−.11]
*Religious and class identity*												
Religious											−.03	[−.08,.03]
Lower class											Reference	
Lower middle class											.04	[−.03,.11]
Middle class											.01	[−.07,.09]
Upper middle class											.09	[−.11,.29]
Upper class											Not estimable	
Observations	968		968		793		775		747		739	
Pseudo R−squared	.075		.078		.236		.250		.268		.275	

*p < 0.05, **p < 0.01, ***p < 0.001

*AME* Average marginal effect, *CI* Confidence intervals

Logistic regression coefficients (change of log-odds ratios) are reported in Table A3 in the Online Appendix

Outcome variable: vaccine hesitancy

Involving COVID-19 experience in Model 2 does not lead to substantial changes, but once fear and precautious behavior indices are involved (Model 3-Model 6), the effects of being woman, opposition voter and 50–59 years old lose significance. The effect size of both being an undecided voter and being older than 60 halves in these models and their significance also reduces with involving more variables. COVID-19 survivors were 13 pp less likely to be hesitant (*p* < 0.05) in Model 2, but it loses significance in Model 3–Model 6.

Model 3 shows that 0.1 points higher COVID-19-fear index decreases the probability of being vaccine hesitant by 5.1 pp on average. 0.1 points higher precautious behavior index decreases this probability by 1.7 pp on average. These effects keep significance and effect sizes only slightly change in Model 4–Model 6. Personal fear index’s effect is insignificant in Model 3, but after involving personal relationship variables, it became significant at *p* < 0.05 (Model 4–Model 6). 0.1 point higher personal fear index results in 1.5–1.7 pp higher probability of vaccine hesitancy on average.

Model 4 reveals that fear from partner’s aggressive behavior during the lockdown is negatively associated with vaccine hesitancy, those who are concerned about it were 9 pp less likely to be hesitant about vaccination (*p* < 0.01). Effect size and significance of this variable slightly increased in Model 5 and Model 6. Relationship status only had significant effect after involving economic variables in the analysis (Model 5 and Model 6). Model 6 shows that childless people living in a relationship were 8 pp more likely to be hesitant than childless, single people (*p* < 0.05).

Model 5 reveals that economic stability increases vaccine acceptance. Regarding dynamic economic evaluation, those who perceived that their financial situation worsened were 6 pp more (*p* < 0.05), while those who experienced financial improvements were 15 pp less likely to be hesitant (*p* < 0.01), both groups compared to those experiencing no change. Amongst groups based on static economic evaluation, those groups who were better off were more likely to accept vaccination compared to those whose income barely covered living expenditures. Those who had no financial problems, but could not save up were 8 pp, those who could save up a little were 13 pp and those who could save much were 19 pp less likely to be vaccine hesitant. Model 6 showed that religiousness and class identity did not significantly influence willingness to vaccinate.

Table 5 shows that all kinds of pandemic-related conspiratorial beliefs significantly correlate with vaccine hesitancy. In the first set of models, belief in “No virus theory” has the strongest effect, 0.1 point increase results in 3.8 pp higher probability of vaccine hesitancy. The effect size of “Population control theory” and “Microchip theory” are somewhat smaller (3.2 pp and 2.7 pp). “China theory” and “Pharma theory” have the weakest effects (2 pp, 2.3 pp). All conspiracy measures’ effects were significant at *p* < 0.001 in these models.

**Table 5:** Conspiratorial beliefs’ effects on the probability of vaccine hesitancy, weighted logistic regression models

	Model 7		Model 8		Model 9		Model 10		Model 11	
VARIABLES	AME	95% CI	AME	95% CI	AME	95% CI	AME	95% CI	AME	95% CI
Conspiracy theory	There is no virus		China intentionally infected the world		Pharma companies created the virus		Microchip in the vaccines		Vaccination is population control	
Conspiratorial belief (0–1 scale)	0.38***	[0.30; 0.47]	0.20***	[0.12; 0.29]	0.23***	[0.15; 0.31]	0.27***	[0.18; 0.35]	0.32***	[0.23; 0.41]
Control variables	Yes		Yes		Yes		Yes		Yes	
Observations	917		844		854		848		714	
Pseudo R-squared	0.140		0.089		0.086		0.113		0.119	

	Model 12		Model 13		Model 14		Model 15		Model 16	
VARIABLES	AME	95% CI	AME	95% CI	AME	95% CI	AME	95% CI	AME	95% CI
Conspiracy theory	There is no virus		China intentionally infected the world		Pharma companies created the virus		Microchip in the vaccines		Vaccination is population control	
Non-believer (1–3)	Reference		Reference		Reference		Reference		Reference	
Uncertain (4–7)	0.20***	[0.13; 0.28]	0.04	[−0.02; 0.10]	0.07*	[0.01; 0.13]	0.08*	[0.01; 0.16]	0.15***	[0.08; 0.22]
Believer (8–10)	0.33***	[0.19; 0.48]	0.20***	[0.11; 0.28]	0.19***	[0.10; 0.27]	0.30***	[0.16; 0.45]	0.26***	[0.13; 0.39]
Do not know/Refuse to answer	0.23***	[0.15; 0.31]	0.15***	[0.07; 0.24]	0.23***	[0.14; 0.32]	0.21***	[0.12; 0.30]	0.11***	[0.04; 0.17]
Control variables	Yes		Yes		Yes		Yes		Yes	
Observations	968		968		968		968		968	
Pseudo R-squared	0.138		0.105		0.112		0.115		0.108	

*p < 0.05, **p < 0.01, ***p < 0.001

*AME* Average marginal effect, *CI* Confidence intervals

Full models and logistic regression coefficients (change of log-odds ratios) are reported in Tables A4 and A5 in the Online Appendix

Control variables are demographic and political characteristics, COVID-19 experience

Outcome variable: vaccine hesitancy

In the second set of models, the groups of non-believers of given theories serve as baselines. People hesitant about “No virus theory” and “Population control theory” were 20 pp and 15 pp more likely to be vaccine hesitant (*p* < 0.001). Respondents hesitant about “Pharma theory” and “Microchip theory” were 7 pp and 8 pp more likely to be vaccine hesitant (*p* < 0.05), but people hesitant about “China theory” did not differ significantly. Believers of all theories had lower probability to accept vaccination (*p* < 0.001). The effects of being believer of “No virus theory” and “Microchip theory” were particularly strong (33 pp and 30 pp higher probability of vaccine hesitancy). Believers of Population control, China and Pharma theories are 26, 20 and 19 pp more likely to be hesitant than non-believers. Respondents not expressing their views were also significantly more likely to be vaccine hesitant regarding all theories (*p* < 0.001).

## Conclusion and discussion

This article presents descriptive statistics about vaccine hesitancy based on data collected in March 2021, and the results of exploratory multivariate analysis to identify the major individual-level determinants of vaccine hesitancy. We developed a multi-item survey measurement of vaccine hesitancy, since we assumed that it gives a more reliable metric for vaccine hesitancy than the usually used single-question based variables. We believe that it is a further proof for the validity of our measurement that 26% of the adult Hungarian population was vaccine hesitant in March 2021 based on our weighted survey data, and the ratio of unvaccinated adults is 27% almost one year later (European Centre for Disease Prevention and Control, 2022b).

In accordance with several previous research studies about COVID-19 vaccine hesitancy (see the meta-analysis of Robinson et al., 2021), our research revealed that older people and women had a higher probability of accepting vaccination. Although previous research presented that the residence (living in rural areas, see Gerretsen et al., 2021) and level of education have significant effects (Gerretsen et al., 2021; Robinson et al., 2021; Troiano & Nardi, 2021), these variables were not statistically significant predictors of vaccine hesitancy in our research. When it is compared to government supporters, the probability of being vaccine hesitant is higher amongst opposition voters in Hungary. Undecided voters are the most likely not to accept vaccination: the effect of being politically non-affiliated is many times higher than the effect size of being an opposition supporter. We can find different, complementary explanations for this phenomenon. Recent studies showed that COVID-19 vaccine hesitancy is associated with the lack of trust (Gerretsen et al., 2021; Dabla-Norris et al., 2021; Troiano & Nardi, 2021; Freeman et al., 2020; Murphy et al., 2021), and undecided voters score lower on trust scales (see evidence from the US: Wilkes, 2015). Both the Hungarian government parties and the united opposition encouraged people to get vaccinated. It is a valid assumption that undecided voters were more resistant to public health communication by political actors than the supporters of established parties. The direction of causation works the other way as well: vaccine hesitant partisans could get disappointed in their political camp.

On one hand, precautiousness (to what extent respondents followed various behaviors to prevent catching and spreading COVID-19) and fear from the illness increases the probability of vaccine acceptance. On the other hand, personal fears (average level of fears from non-pandemic related situations) are positively associated with vaccine hesitancy. Our analysis also showed that COVID-19 survivors were also less likely to be vaccine hesitant, but similarly to political preference and age, this variable lost significance and effect size once we controlled for personal and COVID-19-related fears, precautious behavior. These variables – to some extent – seem to be the mediators of the effect of age, political affiliation and COVID-19 survival. In other words, younger people, undecided voters and those who did not catch COVID-19 have a higher probability of not intending to get vaccinated to a large extent because they might take the virus less seriously, or because they are more nervous in general (not pandemic-wise).

The effect of economic status should be also emphasized. Similarly to age’s effect, this finding is also consistent with recent COVID-19 vaccine hesitancy studies (Gerretsen et al., 2021; Robinson et al., 2021; Troiano & Nardi, 2021). Higher level of financial security comes along with lower probability of vaccine hesitancy regardless of the individual’s level of education, or class identity. In other words, our current economic, social and public health crisis is deeply interconnected. A highly important, unique finding of our research is that vaccination can be a key to the exit door from toxic relationships for many, as people who feared from their romantic partner’s aggression or even experienced domestic violence during the lockdown, were more likely to accept the jab. On the other hand, one model showed that childless couples are more likely to be vaccine hesitant than single, childless people suggesting that couples may reinforce their doubts about vaccination in each other – possibly including fears about vaccination’s adverse effects on fertility.

We examined the effect of this and other pandemic-related false beliefs. The acceptance of all examined conspiracy theories positively correlates with vaccine hesitancy. The effects of beliefs in three theories on the probability of being vaccine hesitant are particularly strong. These are the “microchip in the vaccine” and “vaccination is population control” theories and virus denial. Theories giving alternative explanations for the origin of the virus – “China intentionally infected the world” and “Pharma companies developed the virus” – have somewhat smaller effects on the probability of being vaccine hesitant. These results join to the growing body of literature advocating the importance of developing innovative techniques debunking COVID-19-related misinformation to overcome wide-scale vaccine-hesitancy. Based on our results, we would like to point out that these messages should be primarily targeted to young people, undecided voters and the economically disadvantaged.

## Supplementary Information

Below is the link to the electronic supplementary material.

Supplementary file1 (DOCX 59 kb)

Supplementary file2 (DOCX 28 kb)

## Data Availability

Data are available on the Open Science Framework at the following link: https://osf.io/dbqs8/

## Code availability

The codes to reproduce the analyses presented in the current research is available on the Open Science Framework at the following link: https://osf.io/sm5b4/
