# Supplementary material for: The roots of COVID-19 vaccine hesitancy: evidence from Hungary
Source: J Behav Med. 2022 May 14;46(1-2):185–200. doi: 10.1007/s10865-022-00314-5 (PMC9106981; doi:10.1007/s10865-022-00314-5)
Supplement: Supplementary file 1 — Supplementary file1 (DOCX 59 kb) [file 10865_2022_314_MOESM1_ESM.docx]

**ONLINE SUPPLEMENTARY MATERIAL 1: ONLINE APPENDIX**

**Table A1 Components of indices used as independent variables**

|  | Component questions | Survey question measurement |
| --- | --- | --- |
| Personal fear index | (1) People with different cultural background, migrants move to your neighborhood.  (2) Somebody from your family moves abroad.  (3) You become vulnerable and lose control over your fate.  (4) The world is accelerating even faster; you are unable to catch up with the constant changes.  (5) You get seriously ill, hospitalized  (6) Your relationship breaks up or you cannot find a partner  (7) Your loved ones get hurt  (8) Your financial situation becomes unstable; you will not be able to pay your bills  (9) You become indebted and have to get loans  (10) You lose your home, become homeless  (11) You become the victim of school or workplace discrimination  (12) You become a victim of crime, violent attack | 5-point scale  (1 – Does not evoke fear at all, 5 – Evoke strong fear) |
| COVID-19 fear index | (1) In your opinion, how serious threat does coronavirus pose to human health?  (2) If you got infected with coronavirus, how serious would its consequences be?  (3) Overall, to what extent do you consider coronavirus a serious threat to the health of your family? (4) How much do you fear that coronavirus infection causes serious, long-term complications even after the acute course of the illness? | 4-point scale (1 – Not at all, 2 – Rather not,  3 – Rather yes, 4 – A lot) |
| Pre-cautious behavior index | How often do you follow the behaviors below to prevent coronavirus infection?  (1) If you are outside your home, you are wearing a face mask.  (2) If you leave your home, whenever you have a chance to, you disinfect your hands, wash your hands with soap.  (3) If you are outside your home, you will keep at least 2 meters of distance when talking to others and waiting in line.  (4) Avoid public transportation as much as possible.  (5) When you meet friends, acquaintances, co-workers, you avoid physical contact (handshake, hug, welcome kiss).  (6) You cancel or avoid gatherings where more people would have been expected to attend.  (7) You avoid contact with persons (e.g.: elderly and/ or chronic patients) to whom being infected with coronavirus is particularly risky.  (8) You only leave home for the most necessary reasons.  (9) You take vitamins to strengthen your immune system. | 4-point scale (1 - Not at all, 2 – Sometimes, 3 – Mostly, 4 – Always) |
| Social isolation index | In what follows, we will ask whether your human relationships have become closer, or became distant during the period of quarantine and social distancing?  (1) Family relationships  (2) Friendships | 5-point scale (1 – Became much closer, 2 – Became closer, 3 – Did not change, 4 – Became distant, 5 Became totally distant) |

*Note: The table is based on translations from the original Hungarian questionnaire*

**Table A2 Coding of further exploratory variables**

|  | **Component questions** | **Survey question measurement and recoding** |
| --- | --- | --- |
| COVID survivor | People are worried about coronavirus and its consequences for several reasons. How worried are you about the following? Is there anything that has already happened? You get sick | COVID-survivor: 5 – Already happened  Not COVID-survivor: all other answers, ND/NA |
| Relationship status | Do you have a child under the age of 18 with whom you live in the same household?  Do you have a partner with whom you live in the same household? | Single no child: No, No  In a relationship, no child: No, Yes  Single parent: Yes, No  In a relationship, with child: Yes, Yes |
| Fear from partner | People are worried about coronavirus and its consequences for several reasons. How worried are you about the following? Is there anything that has already happened?  Your partner becomes nervous, aggressive during home confinement (only asked from respondents living with their partner) | Fears from partner: 3 – Rather worrying, 4 – Seriously worried, 8 – Has already happened  Does not fear from partner: 1 – Not worried at all, 2 – Rather not worried, ND/NA |
| Financial status (dynamic) | Did the financial situation of your household improve or worsen since the outbreak of the coronavirus epidemic in Hungary in March 2020? | No recoding, ND/NA excluded:  1 – Worsened  2 – Did not change  3 – Improved |
| Financial status (static) | Which one of these statements describe the best your household’s current status? | No recoding, ND/NA excluded:  1 – We are indebted, we live on loans or external aid  2 – We are living off our previous savings  3 – Our income barely covers living costs  4 – We have no financial problems, but cannot save up  5 – We have no financial problems, and we can save up a little amount of money  6 – We have no financial problems, and we can save up significant amount of money |
| Religiousness | Which one of these statements describe you the most? | Religious: 1 – I am religious, I follow the teachings of the church, 2 – I am religious in my own way Not religious: 3 – I cannot say whether I am religious or not, 4 – I am not religious, 5 – I have different world view, confidently, ND/NA |
| Class identity | In which social class would you classify yourself? | No recoding, ND/NA excluded: 1 – Lower class  2 – Lower middle class  3 – Middle class  4 – Upper middle class  5 – Upper class |

*Note: The table is based on translations from the original Hungarian questionnaire*

**Table A3 Political, demographic, social, economic roots of vaccine hesitancy, weighted logistic regression models – Logistic regression coefficients (Average marginal effects are reported in Table 1 of the article)**

|  | Model 1 | | Model 2 | | Model 3 | | Model 4 | | Model 5 | | Model 6 | |
| --- | --- | --- | --- | --- | --- | --- | --- | --- | --- | --- | --- | --- |
| VARIABLES | **AME** | **95% CI** | **AME** | **95% CI** | **AME** | **95% CI** | **AME** | **95% CI** | **AME** | **95% CI** | **AME** | **95% CI** |
| Political preferences |  |  |  |  |  |  |  |  |  |  |  |  |
| Government voter | Reference | | Reference | | Reference | | Reference | | Reference | | Reference | |
| Opposition voter | 0.63** | [0.25,1.01] | 0.62** | [0.25,1.00] | 0.24 | [-0.23,0.70] | 0.17 | [-0.31,0.65] | 0.12 | [-0.38,0.63] | 0.04 | [-0.48,0.57] |
| Undecided voter | 1.09*** | [0.70,1.49] | 1.11*** | [0.71,1.52] | 0.79** | [0.29,1.30] | 0.77** | [0.25,1.29] | 0.66* | [0.11,1.21] | 0.59* | [0.03,1.15] |
| **Demographic variables** | | | | | | | | | | | | |
| Elementary school or less | Reference | | Reference | | Reference | | Reference | | Reference | | Reference | |
| Vocational school | -0.11 | [-0.55,0.34] | -0.10 | [-0.55,0.35] | -0.11 | [-0.67,0.45] | -0.12 | [-0.69,0.45] | 0.09 | [-0.49,0.67] | -0.01 | [-0.64,0.62] |
| Secondary school | -0.16 | [-0.58,0.27] | -0.17 | [-0.60,0.26] | 0.10 | [-0.44,0.65] | 0.18 | [-0.37,0.73] | 0.43 | [-0.18,1.03] | 0.40 | [-0.28,1.07] |
| Higher education | -0.41 | [-0.89,0.08] | -0.39 | [-0.88,0.09] | -0.47 | [-1.08,0.15] | -0.34 | [-0.96,0.27] | 0.05 | [-0.69,0.79] | -0.04 | [-0.90,0.82] |
| 18-29 | Reference | | Reference | | Reference | | Reference | | Reference | | Reference | |
| 30-39 | -0.21 | [-0.68,0.26] | -0.21 | [-0.68,0.26] | 0.08 | [-0.53,0.69] | 0.14 | [-0.51,0.79] | 0.08 | [-0.61,0.76] | 0.06 | [-0.64,0.76] |
| 40-49 | -0.29 | [-0.76,0.19] | -0.27 | [-0.74,0.21] | -0.07 | [-0.67,0.54] | -0.06 | [-0.73,0.61] | 0.00 | [-0.69,0.69] | 0.01 | [-0.70,0.71] |
| 50-59 | -0.58* | [-1.07,-0.09] | -0.57* | [-1.06,-0.07] | -0.25 | [-0.85,0.36] | -0.25 | [-0.90,0.40] | -0.20 | [-0.87,0.47] | -0.25 | [-0.94,0.45] |
| 60 or older | -1.35*** | [-1.86,-0.83] | -1.36*** | [-1.87,-0.84] | -0.83* | [-1.50,-0.16] | -0.84* | [-1.53,-0.15] | -0.85* | [-1.59,-0.10] | -0.81* | [-1.57,-0.06] |
| Budapest | Reference | | Reference | | Reference | | Reference | | Reference | | Reference | |
| County seats | 0.40 | [-0.12,0.93] | 0.41 | [-0.11,0.94] | 0.23 | [-0.43,0.90] | 0.26 | [-0.44,0.97] | 0.33 | [-0.43,1.09] | 0.35 | [-0.41,1.12] |
| Towns | 0.03 | [-0.44,0.49] | 0.06 | [-0.40,0.53] | -0.02 | [-0.64,0.60] | 0.03 | [-0.62,0.69] | 0.18 | [-0.51,0.87] | 0.20 | [-0.49,0.90] |
| Villages | 0.04 | [-0.44,0.51] | 0.05 | [-0.43,0.52] | 0.32 | [-0.30,0.94] | 0.21 | [-0.44,0.87] | 0.29 | [-0.40,0.98] | 0.29 | [-0.40,0.98] |
| Male | Reference | | Reference | | Reference | | Reference | | Reference | | Reference | |
| Female | -0.34* | [-0.65,-0.03] | -0.33* | [-0.64,-0.01] | -0.30 | [-0.69,0.10] | -0.32 | [-0.72,0.09] | -0.32 | [-0.74,0.10] | -0.33 | [-0.76,0.10] |
| **COVID-19 experience** | | | | | | | | | | | | |
| COVID-19 survivor |  |  | -0.91 | [-1.97,0.14] | -0.51 | [-1.77,0.75] | -0.63 | [-1.87,0.61] | -0.41 | [-1.64,0.81] | -0.34 | [-1.55,0.87] |
| **Fear and precaution indices** | | | | | | | | | | | | |
| Personal fear index (0-1) |  |  |  |  | 0.93 | [-0.03,1.88] | 1.33* | [0.29,2.38] | 1.23* | [0.13,2.34] | 1.21* | [0.07,2.36] |
| Covid-19 fear index (0-1) |  |  |  |  | -3.87*** | [-4.82,-2.92] | -3.93*** | [-4.87,-2.98] | -4.03*** | [-5.02,-3.04] | -4.10*** | [-5.12,-3.08] |
| Precautious behavior index (0-1) |  |  |  |  | -1.31* | [-2.31,-0.30] | -1.43** | [-2.46,-0.41] | -1.53** | [-2.61,-0.45] | -1.42* | [-2.52,-0.31] |

Table continues on next page

| **Personal relationships** | | | | | | | | | | | | |
| --- | --- | --- | --- | --- | --- | --- | --- | --- | --- | --- | --- | --- |
| Single. no children |  |  |  |  | Reference | | Reference | | Reference | | Reference | |
| In a relationship. no children |  |  |  |  |  |  | 0.46 | [-0.13,1.06] | 0.60 | [-0.03,1.23] | 0.64 | [-0.01,1.29] |
| Single parent |  |  |  |  |  |  | -0.95 | [-2.31,0.40] | -1.16 | [-2.61,0.29] | -1.09 | [-2.55,0.37] |
| In a relationship. with children |  |  |  |  |  |  | 0.33 | [-0.29,0.95] | 0.40 | [-0.26,1.07] | 0.45 | [-0.22,1.12] |
| Social isolation index (0-1) |  |  |  |  |  |  | 1.02 | [-0.47,2.50] | 0.63 | [-0.86,2.13] | 0.61 | [-0.95,2.17] |
| Fear from partner |  |  |  |  |  |  | -0.71** | [-1.24,-0.19] | -0.84** | [-1.38,-0.29] | -0.91** | [-1.47,-0.36] |
| **Economic status** | | | | | | | | | | | | |
| Fin. situation did not change |  |  |  |  |  |  | Reference | | Reference | | Reference | |
| Fin. situation worsened |  |  |  |  |  |  |  |  | 0.44* | [0.00,0.88] | 0.49* | [0.04,0.93] |
| Fin. situation improved |  |  |  |  |  |  |  |  | -1.88 | [-3.84,0.09] | -1.97* | [-3.88,-0.05] |
| Living on loans/aid |  |  |  |  |  |  |  |  | -0.67 | [-1.60,0.26] | -0.57 | [-1.51,0.36] |
| Live off savings |  |  |  |  |  |  |  |  | -0.29 | [-0.95,0.36] | -0.26 | [-0.91,0.39] |
| Income barely covers living |  |  |  |  |  |  |  |  | Reference | | Reference | |
| No financial problem. but cannot save up |  |  |  |  |  |  |  |  | -0.65* | [-1.23,-0.08] | -0.66* | [-1.25,-0.08] |
| Can save up a little |  |  |  |  |  |  |  |  | -1.14* | [-2.02,-0.27] | -1.12* | [-2.05,-0.20] |
| Can save up significant amount |  |  |  |  |  |  |  |  | -1.96* | [-3.57,-0.34] | -2.25* | [-4.00,-0.50] |
| **Religious and class identity** | | | | | | | | | | | | |
| Religious |  |  |  |  |  |  |  |  |  |  | -0.20 | [-0.65,0.24] |
| Lower class |  |  |  |  |  |  |  |  |  |  | Reference | |
| Lower middle class |  |  |  |  |  |  |  |  |  |  | 0.35 | [-0.22,0.91] |
| Middle class |  |  |  |  |  |  |  |  |  |  | 0.11 | [-0.56,0.79] |
| Upper middle class |  |  |  |  |  |  |  |  |  |  | 0.70 | [-0.73,2.12] |
| Upper class |  |  |  |  |  |  |  |  |  |  | Not estimable | |
| Constant | -0.92** | [-1.56,-0.27] | -0.92** | [-1.57,-0.28] | 1.77** | [0.59,2.96] | 1.13 | [-0.29,2.56] | 1.32 | [-0.20,2.84] | 1.27 | [-0.25,2.79] |
| Observations | 968 | | 968 | | 793 | | 775 | | 747 | | 739 | |
| Pseudo R-squared | 0.075 | | 0.078 | | 0.236 | | 0.250 | | 0.268 | | 0.275 | |

*Note: * p<0.05, ** p<0.01, *** p<0.001
Betas show change in log-odds ratios, CI: Confidence intervals
Outcome variable: vaccine hesitancy*

**Table A4 Conspiratorial beliefs and vaccine hesitancy, weighted logistic regression models, outcome variable: vaccine hesitancy – Average marginal effects (Full models, shortened form of the models are presented in Table 2 of the article)**

|  | Model 7 | | Model 8 | | Model 9 | | Model 10 | | Model 11 | |
| --- | --- | --- | --- | --- | --- | --- | --- | --- | --- | --- |
| **VARIABLES** | **AME** | **95% CI** | **AME** | **95% CI** | **AME** | **95% CI** | **AME** | **95% CI** | **AME** | **95% CI** |
| Conspiracy theory | There is no virus | | China intentionally infected the world | | Pharma companies created the virus | | Microchip in the vaccines | | Vaccination is population control | |
|  |  |  |  |  |  |  |  |  |  |  |
| Conspiratorial belief (0-1 scale) | 0.38*** | [0.30; 0.47] | 0.20*** | [0.12; 0.29] | 0.23*** | [0.15; 0.31] | 0.27*** | [0.18; 0.35] | 0.32*** | [0.23; 0.41] |
|  |  |  |  |  |  |  |  |  |  |  |
| **Political preferences** | | | | | | | | | | |
| Government voter | Reference | | Reference | | Reference | | Reference | | Reference | |
| Opposition voter | 0.07* | [0.01; 0.13] | 0.07* | [0.01; 0.13] | 0.06* | [0.00; 0.12] | 0.05 | [-0.01; 0.11] | 0.05 | [-0.01; 0.12] |
| Undecided voter | 0.14*** | [0.07; 0.21] | 0.17*** | [0.09; 0.24] | 0.13*** | [0.05; 0.20] | 0.14*** | [0.06; 0.21] | 0.13** | [0.04; 0.21] |
| **Demographic variables** | | | | | | | | | | |
| Elementary school or less | Reference | | Reference | | Reference | | Reference | | Reference | |
| Vocational school | -0.02 | [-0.10; 0.06] | -0.03 | [-0.11; 0.06] | -0.01 | [-0.09; 0.07] | -0.03 | [-0.12; 0.05] | -0.04 | [-0.14; 0.05] |
| Secondary school | -0.04 | [-0.11; 0.04] | 0 | [-0.08; 0.09] | -0.01 | [-0.09; 0.06] | -0.04 | [-0.12; 0.04] | -0.02 | [-0.11; 0.06] |
| Higher education | -0.06 | [-0.14; 0.02] | -0.03 | [-0.12; 0.06] | -0.03 | [-0.12; 0.05] | -0.05 | [-0.14; 0.03] | -0.03 | [-0.13; 0.06] |
| 18-29 | Reference | | Reference | | Reference | | Reference | | Reference | |
| 30-39 | -0.04 | [-0.14; 0.05] | -0.01 | [-0.11; 0.10] | -0.03 | [-0.13; 0.07] | 0.02 | [-0.08; 0.12] | 0.03 | [-0.08; 0.14] |
| 40-49 | -0.06 | [-0.15; 0.04] | -0.04 | [-0.14; 0.06] | -0.08 | [-0.18; 0.02] | -0.03 | [-0.13; 0.07] | -0.05 | [-0.15; 0.06] |
| 50-59 | -0.12** | [-0.22; -0.03] | -0.08 | [-0.17; 0.02] | -0.08 | [-0.18; 0.01] | -0.08 | [-0.17; 0.02] | -0.06 | [-0.16; 0.04] |
| 60 or older | -0.23*** | [-0.31; -0.15] | -0.18*** | [-0.27; -0.09] | -0.19*** | [-0.28; -0.11] | -0.20*** | [-0.29; -0.12] | -0.17*** | [-0.26; -0.07] |
| Budapest | Reference | | Reference | | Reference | | Reference | | Reference | |
| County seats | 0.08 | [-0.01; 0.17] | 0.06 | [-0.04; 0.16] | 0.05 | [-0.05; 0.15] | 0.06 | [-0.03; 0.16] | 0.09 | [-0.01; 0.20] |
| Towns | -0.01 | [-0.09; 0.06] | -0.02 | [-0.10; 0.06] | 0 | [-0.08; 0.08] | -0.02 | [-0.10; 0.06] | -0.02 | [-0.10; 0.07] |
| Villages | -0.01 | [-0.09; 0.07] | -0.03 | [-0.11; 0.05] | -0.02 | [-0.11; 0.06] | -0.02 | [-0.10; 0.06] | -0.01 | [-0.10; 0.07] |
| Male | Reference | | Reference | | Reference | | Reference | | Reference | |
| Female | -0.03 | [-0.08; 0.02] | -0.03 | [-0.09; 0.02] | -0.03 | [-0.08; 0.03] | -0.03 | [-0.09; 0.02] | -0.04 | [-0.10; 0.02] |
| **COVID-19 experience** | | | | | | | | | | |
| COVID-19 survivor | -0.13** | [-0.22; -0.03] | -0.09 | [-0.22; 0.03] | -0.11 | [-0.23; 0.01] | -0.1 | [-0.21; 0.01] | -0.09 | [-0.22; 0.04] |
|  |  |  |  |  |  |  |  |  |  |  |
| Observations | 917 | | 844 | | 854 | | 848 | | 714 | |
| Pseudo R-squared | 0.140 | | 0.089 | | 0.086 | | 0.113 | | 0.119 | |

Table continues on next page

|  | Model 12 | | Model 13 | | Model 14 | | Model 15 | | Model 16 | |
| --- | --- | --- | --- | --- | --- | --- | --- | --- | --- | --- |
| **VARIABLES** | **AME** | **95% CI** | **AME** | **95% CI** | **AME** | **95% CI** | **AME** | **95% CI** | **AME** | **95% CI** |
| Conspiracy theory | There is no virus | | China intentionally infected the world | | Pharma companies created the virus | | Microchip in the vaccines | | Vaccination is population control | |
|  |  |  |  |  |  |  |  |  |  |  |
| Not believer (1-3) | Reference | | Reference | | Reference | | Reference | | Reference | |
| Uncertain (4-7) | 0.20*** | [0.13; 0.28] | 0.04 | [-0.02; 0.10] | 0.07* | [0.01; 0.13] | 0.08* | [0.01; 0.16] | 0.15*** | [0.08; 0.22] |
| Believer (8-10) | 0.33*** | [0.19; 0.48] | 0.20*** | [0.11; 0.28] | 0.19*** | [0.10; 0.27] | 0.30*** | [0.16; 0.45] | 0.26*** | [0.13; 0.39] |
| Do not know/Refuse to answer | 0.23*** | [0.15; 0.31] | 0.15*** | [0.07; 0.24] | 0.23*** | [0.14; 0.32] | 0.21*** | [0.12; 0.30] | 0.11*** | [0.04; 0.17] |
|  |  |  |  |  |  |  |  |  |  |  |
| **Political preferences** | | | | | | | | | | |
| Government voter | Reference | | Reference | | Reference | | Reference | | Reference | |
| Opposition voter | 0.08** | [0.02; 0.14] | 0.08** | [0.03; 0.14] | 0.09** | [0.03; 0.15] | 0.08** | [0.02; 0.14] | 0.08** | [0.02; 0.14] |
| Undecided voter | 0.15*** | [0.08; 0.21] | 0.18*** | [0.11; 0.25] | 0.16*** | [0.10; 0.23] | 0.16*** | [0.09; 0.23] | 0.16*** | [0.09; 0.24] |
| **Demographic variables** | | | | | | | | | | |
| Elementary school or less | Reference | | Reference | | Reference | | Reference | | Reference | |
| Vocational school | 0 | [-0.07; 0.08] | -0.01 | [-0.09; 0.06] | 0 | [-0.08; 0.08] | -0.01 | [-0.09; 0.07] | -0.01 | [-0.09; 0.06] |
| Secondary school | -0.01 | [-0.08; 0.06] | 0 | [-0.08; 0.07] | -0.01 | [-0.09; 0.06] | -0.01 | [-0.09; 0.06] | -0.02 | [-0.09; 0.05] |
| Higher education | -0.04 | [-0.12; 0.04] | -0.04 | [-0.12; 0.04] | -0.04 | [-0.12; 0.04] | -0.04 | [-0.12; 0.04] | -0.05 | [-0.13; 0.03] |
| 18-29 | Reference | | Reference | | Reference | | Reference | | Reference | |
| 30-39 | -0.05 | [-0.14; 0.04] | -0.04 | [-0.14; 0.05] | -0.05 | [-0.14; 0.05] | -0.04 | [-0.13; 0.06] | -0.03 | [-0.13; 0.06] |
| 40-49 | -0.07 | [-0.16; 0.02] | -0.05 | [-0.14; 0.05] | -0.07 | [-0.16; 0.03] | -0.05 | [-0.14; 0.05] | -0.04 | [-0.14; 0.05] |
| 50-59 | -0.12** | [-0.21; -0.03] | -0.10* | [-0.20; -0.01] | -0.11* | [-0.20; -0.01] | -0.10* | [-0.20; -0.01] | -0.10* | [-0.20; -0.01] |
| 60 or older | -0.23*** | [-0.31; -0.15] | -0.21*** | [-0.30; -0.13] | -0.22*** | [-0.31; -0.14] | -0.22*** | [-0.30; -0.14] | -0.21*** | [-0.29; -0.13] |
| Budapest | Reference | | Reference | | Reference | | Reference | | Reference | |
| County seats | 0.08 | [-0.01; 0.17] | 0.07 | [-0.02; 0.16] | 0.07 | [-0.02; 0.17] | 0.08 | [-0.01; 0.17] | 0.09 | [-0.01; 0.18] |
| Towns | 0 | [-0.07; 0.08] | 0 | [-0.08; 0.07] | 0.01 | [-0.07; 0.09] | 0.01 | [-0.07; 0.09] | 0.01 | [-0.06; 0.09] |
| Villages | 0.01 | [-0.07; 0.08] | -0.01 | [-0.09; 0.07] | 0 | [-0.08; 0.08] | 0.02 | [-0.06; 0.10] | 0.02 | [-0.05; 0.10] |
| Male | Reference | | Reference | | Reference | | Reference | | Reference | |
| Female | -0.05* | [-0.11; -0.00] | -0.06* | [-0.11; -0.00] | -0.05 | [-0.10; 0.00] | -0.04 | [-0.10; 0.01] | -0.06* | [-0.11; -0.00] |
| **COVID-19 experience** | | | | | | | | | | |
| COVID-19 survivor | -0.13* | [-0.23; -0.02] | -0.11 | [-0.23; 0.02] | -0.12 | [-0.23; 0.00] | -0.1 | [-0.22; 0.03] | -0.12* | [-0.24; -0.00] |
|  |  |  |  |  |  |  |  |  |  |  |
| Observations | 968 | | 968 | | 968 | | 968 | | 968 | |
| Pseudo R-squared | 0.138 | | 0.105 | | 0.112 | | 0.115 | | 0.108 | |

*Note: * p<0.05, ** p<0.01, *** p<0.001
AME: Average marginal effect, CI: Confidence intervals
Outcome variable: vaccine hesitancy*

**Table A5 Conspiratorial beliefs and vaccine hesitancy, weighted logistic regression models, outcome variable: vaccine hesitancy – Logistic regression coefficients (Full models, shortened form of the models are presented in Table 2 of the article)**

|  | Model 7 | | Model 8 | | Model 9 | | Model 10 | | Model 11 | |
| --- | --- | --- | --- | --- | --- | --- | --- | --- | --- | --- |
| **VARIABLES** | **Beta** | **95% CI** | **Beta** | **95% CI** | **Beta** | **95% CI** | **Beta** | **95% CI** | **Beta** | **95% CI** |
| Conspiracy theory | There is no virus | | China intentionally infected the world | | Pharma companies created the virus | | Microchip in the vaccines | | Vaccination is population control | |
|  |  |  |  |  |  |  |  |  |  |  |
| Conspiratorial belief (0-1 scale) | 2.53*** | [1.91; 3.15] | 1.25*** | [0.73; 1.78] | 1.41*** | [0.88; 1.93] | 1.73*** | [1.15; 2.31] | 2.05*** | [1.42; 2.68] |
|  |  |  |  |  |  |  |  |  |  |  |
| **Political preferences** | | | | | | | | | | |
| Government voter | Reference | | Reference | | Reference | | Reference | | Reference | |
| Opposition voter | 0.50* | [0.08; 0.92] | 0.45* | [0.04; 0.86] | 0.41* | [0.00; 0.82] | 0.34 | [-0.08; 0.76] | 0.36 | [-0.08; 0.80] |
| Undecided voter | 0.89*** | [0.46; 1.33] | 0.98*** | [0.53; 1.42] | 0.78*** | [0.33; 1.22] | 0.85*** | [0.40; 1.29] | 0.76** | [0.26; 1.27] |
| **Demographic variables** | | | | | | | | | | |
| Elementary school or less | Reference | | Reference | | Reference | | Reference | | Reference | |
| Vocational school | -0.13 | [-0.63; 0.37] | -0.16 | [-0.67; 0.35] | -0.07 | [-0.58; 0.45] | -0.21 | [-0.73; 0.31] | -0.28 | [-0.87; 0.32] |
| Secondary school | -0.23 | [-0.72; 0.25] | 0.02 | [-0.46; 0.51] | -0.09 | [-0.57; 0.39] | -0.27 | [-0.76; 0.23] | -0.14 | [-0.67; 0.39] |
| Higher education | -0.39 | [-0.93; 0.14] | -0.21 | [-0.77; 0.36] | -0.22 | [-0.77; 0.33] | -0.34 | [-0.89; 0.21] | -0.21 | [-0.79; 0.38] |
| 18-29 | Reference | | Reference | | Reference | | Reference | | Reference | |
| 30-39 | -0.22 | [-0.72; 0.29] | -0.03 | [-0.55; 0.50] | -0.14 | [-0.67; 0.38] | 0.08 | [-0.43; 0.60] | 0.16 | [-0.41; 0.73] |
| 40-49 | -0.32 | [-0.84; 0.20] | -0.19 | [-0.72; 0.33] | -0.41 | [-0.96; 0.13] | -0.17 | [-0.71; 0.36] | -0.26 | [-0.85; 0.34] |
| 50-59 | -0.72** | [-1.26; -0.18] | -0.41 | [-0.96; 0.14] | -0.46 | [-0.99; 0.07] | -0.44 | [-0.99; 0.11] | -0.34 | [-0.94; 0.25] |
| 60 or older | -1.61*** | [-2.19; -1.02] | -1.18*** | [-1.75; -0.60] | -1.28*** | [-1.83; -0.72] | -1.51*** | [-2.14; -0.88] | -1.16*** | [-1.81; -0.51] |
| Budapest | Reference | | Reference | | Reference | | Reference | | Reference | |
| County seats | 0.46 | [-0.09; 1.02] | 0.33 | [-0.24; 0.89] | 0.30 | [-0.28; 0.88] | 0.37 | [-0.21; 0.96] | 0.53 | [-0.08; 1.14] |
| Towns | -0.10 | [-0.61; 0.41] | -0.12 | [-0.63; 0.38] | -0.01 | [-0.52; 0.51] | -0.13 | [-0.67; 0.41] | -0.12 | [-0.66; 0.43] |
| Villages | -0.07 | [-0.59; 0.45] | -0.19 | [-0.71; 0.34] | -0.15 | [-0.68; 0.38] | -0.15 | [-0.69; 0.39] | -0.08 | [-0.65; 0.48] |
| Male | Reference | | Reference | | Reference | | Reference | | Reference | |
| Female | -0.19 | [-0.54; 0.15] | -0.20 | [-0.55; 0.15] | -0.16 | [-0.51; 0.19] | -0.22 | [-0.57; 0.13] | -0.24 | [-0.62; 0.14] |
| **COVID-19 experience** | | | | | | | | | | |
| COVID-19 survivor | -1.07* | [-2.13; -0.02] | -0.67 | [-1.76; 0.41] | -0.87 | [-2.04; 0.31] | -0.78 | [-1.85; 0.29] | -0.67 | [-1.81; 0.47] |
|  |  |  |  |  |  |  |  |  |  |  |
| Constant | -1.25*** | [-1.97; -0.54] | -1.55*** | [-2.31; -0.79] | -1.49*** | [-2.25; -0.72] | -1.16** | [-1.90; -0.42] | -1.51*** | [-2.30; -0.71] |
| Observations | 917 | | 844 | | 854 | | 848 | | 714 | |
| Pseudo R-squared | 0.140 | | 0.089 | | 0.086 | | 0.113 | | 0.119 | |

Table continues on next page

|  | Model 12 | | Model 13 | | Model 14 | | Model 15 | | Model 16 | |
| --- | --- | --- | --- | --- | --- | --- | --- | --- | --- | --- |
| **VARIABLES** | **Beta** | **95% CI** | **Beta** | **95% CI** | **Beta** | **95% CI** | **Beta** | **95% CI** | **Beta** | **95% CI** |
| Conspiracy theory | There is no virus | | China intentionally infected the world | | Pharma companies created the virus | | Microchip in the vaccines | | Vaccination is population control | |
|  |  |  |  |  |  |  |  |  |  |  |
| Not believer (1-3) | Reference | | Reference | | Reference | | Reference | | Reference | |
| Hesitant (4-7) | 1.17*** | [0.77; 1.56] | 0.28 | [-0.12; 0.68] | 0.46* | [0.07; 0.85] | 0.51* | [0.08; 0.93] | 0.89*** | [0.49; 1.30] |
| Believer (8-10) | 1.76*** | [1.08; 2.45] | 1.10*** | [0.64; 1.56] | 1.09*** | [0.62; 1.55] | 1.54*** | [0.87; 2.21] | 1.42*** | [0.77; 2.06] |
| Do not know/Refuse to answer | 1.29*** | [0.86; 1.72] | 0.90*** | [0.41; 1.39] | 1.27*** | [0.81; 1.73] | 1.11*** | [0.66; 1.56] | 0.67*** | [0.29; 1.06] |
|  |  | |  | |  | |  | |  | |
| **Political preferences** | | | | | | | | | | |
| Government voter | Reference | | Reference | | Reference | | Reference | | Reference | |
| Opposition voter | 0.54** | [0.15; 0.94] | 0.55** | [0.16; 0.94] | 0.58** | [0.19; 0.96] | 0.53** | [0.14; 0.92] | 0.50* | [0.11; 0.89] |
| Undecided voter | 0.90*** | [0.49; 1.31] | 1.05*** | [0.64; 1.47] | 0.97*** | [0.57; 1.38] | 0.97*** | [0.56; 1.39] | 0.96*** | [0.55; 1.37] |
| **Demographic variables** | | | | | | | | | | |
| Elementary school or less | Reference | | Reference | | Reference | | Reference | | Reference | |
| Vocational school | 0.02 | [-0.44; 0.49] | -0.08 | [-0.54; 0.38] | 0.02 | [-0.45; 0.48] | -0.07 | [-0.53; 0.40] | -0.08 | [-0.54; 0.37] |
| Secondary school | -0.06 | [-0.51; 0.40] | -0.02 | [-0.47; 0.42] | -0.09 | [-0.53; 0.35] | -0.09 | [-0.53; 0.36] | -0.12 | [-0.56; 0.32] |
| Higher education | -0.27 | [-0.77; 0.23] | -0.24 | [-0.75; 0.27] | -0.25 | [-0.76; 0.25] | -0.25 | [-0.76; 0.27] | -0.29 | [-0.79; 0.21] |
| 18-29 | Reference | | Reference | | Reference | | Reference | | Reference | |
| 30-39 | -0.26 | [-0.75; 0.22] | -0.20 | [-0.68; 0.28] | -0.24 | [-0.72; 0.24] | -0.19 | [-0.68; 0.29] | -0.17 | [-0.65; 0.32] |
| 40-49 | -0.36 | [-0.85; 0.13] | -0.24 | [-0.72; 0.24] | -0.35 | [-0.83; 0.14] | -0.25 | [-0.73; 0.24] | -0.21 | [-0.68; 0.27] |
| 50-59 | -0.68** | [-1.20; -0.16] | -0.54* | [-1.04; -0.03] | -0.58* | [-1.09; -0.07] | -0.56* | [-1.07; -0.05] | -0.55* | [-1.05; -0.05] |
| 60 or older | -1.50*** | [-2.04; -0.96] | -1.34*** | [-1.86; -0.81] | -1.41*** | [-1.94; -0.89] | -1.42*** | [-1.94; -0.89] | -1.34*** | [-1.86; -0.82] |
| Budapest | Reference | | Reference | | Reference | | Reference | | Reference | |
| County seats | 0.49 | [-0.05; 1.04] | 0.40 | [-0.13; 0.94] | 0.42 | [-0.13; 0.97] | 0.48 | [-0.07; 1.02] | 0.50 | [-0.05; 1.04] |
| Towns | 0.01 | [-0.48; 0.51] | -0.03 | [-0.50; 0.45] | 0.06 | [-0.43; 0.55] | 0.05 | [-0.45; 0.54] | 0.09 | [-0.39; 0.56] |
| Villages | 0.04 | [-0.46; 0.55] | -0.05 | [-0.53; 0.43] | -0.01 | [-0.51; 0.49] | 0.11 | [-0.39; 0.61] | 0.14 | [-0.35; 0.63] |
| Male | Reference | | Reference | | Reference | | Reference | | Reference | |
| Female | -0.33* | [-0.66; -0.01] | -0.34* | [-0.66; -0.02] | -0.31 | [-0.63; 0.01] | -0.27 | [-0.59; 0.05] | -0.34* | [-0.65; -0.02] |
| **COVID-19 experience** | | | | | | | | | | |
| COVID-19 survivor | -0.99 | [-2.04; 0.07] | -0.76 | [-1.85; 0.32] | -0.84 | [-1.88; 0.21] | -0.69 | [-1.75; 0.36] | -0.87 | [-1.95; 0.22] |
|  |  |  |  |  |  |  |  |  |  |  |
| Constant | -1.35*** | [-2.06; -0.64] | -1.35*** | [-2.06; -0.65] | -1.42*** | [-2.14; -0.70] | -1.31*** | [-2.01; -0.61] | -1.45*** | [-2.14; -0.76] |
| Observations | 968 | | 968 | | 968 | | 968 | | 968 | |
| Pseudo R-squared | 0.138 | | 0.105 | | 0.112 | | 0.115 | | 0.108 | |

*Note: * p<0.05, ** p<0.01, *** p<0.001
Betas show change in log-odds ratios, CI: Confidence intervals
Outcome variable: vaccine hesitancy*
