# Supplementary material for: The roots of COVID-19 vaccine hesitancy: evidence from Hungary
Source: J Behav Med. 2022 May 14;46(1-2):185–200. doi: 10.1007/s10865-022-00314-5 (PMC9106981; doi:10.1007/s10865-022-00314-5)
Supplement: Supplementary file 2 — Supplementary file2 (DOCX 28 kb) [file 10865_2022_314_MOESM2_ESM.docx]

**ONLINE SUPPLEMENTARY MATERIAL 2: SURVEY INFORMATION**

The following document consists of the invitation letter, the survey questionnaire (questions used in the analysis of our article “The roots of COVID-19 vaccine hesitancy: evidence from Hungary”) and the declaration of data management. The original versions of these documents (written in Hungarian) are available at the following link: <https://osf.io/tb9vp/>

**INVITATION LETTER**

(provided by our partner, Závecz Research, translated from Hungarian)

Dear Madam/Sir,

We would like you to participate in a public opinion research, in which people can share their opinions about important social problems.

For our request, the colleagues of H-Building Kft. carry out the research, they will show you their identification cards for your request.

Completion of the survey takes 45-50 minutes.

Your response will not be linked to you personally, the aggregate results will be analysed.

We would like to thank you for helping our work!

Budapest, 1 March 2021

Tibor Závecz, executive director

ZRI Závecz Research Research Institute

**Questionnaire**

**(survey questions used in the analyses, translated from Hungarian)**

**Beyond standard demographic* issues:**

**Which one of these statements describe the best your household’s current status? ***

1 – We are indebted, we live on loans or external aid

2 – We are living off our previous savings

3 – Our income barely covers living costs

4 – We have no financial problems, but cannot save up

5 – We have no financial problems, and we can save up a little amount of money

6 – We have no financial problems, and we can save up significant amount of money

**Which one of these statements describe you the most?***

1 – I am religious, I follow the teachings of the church

2 – I am religious in my own way

3 – I cannot say whether I am religious or not

4 – I am not religious

5 – I have different world view, confidently

**In which social class would you classify yourself?***

1 – Lower class

2 – Lower middle class

3 – Middle class

4 – Upper middle class

5 – Upper class

|  | Yes | No | Does not know/ does not want to answer |
| --- | --- | --- | --- |
| **D1) Do you have a child under the age of 18 with whom you live in the same household?** | 1 | 2 | 99 |
| **D2) Are you currently actively employed?** | 1 | 2 | 99 |
| **D3) Do you have a partner with whom you live in the same household?** | 1 | 2 | 99 |

*Standard demographic questions and the questions marked with a star were not written by the authors. These questions are asked in the omnibus questionnaires of Závecz Research on a regular basis.

**Pandemic-related questions:**

**1) In the following, 1 will list some topics that can arouse fear in some people. Please indicate on a scale of 1 to 5, according to the classification used in schools, the extent to which the following things arouse fear in you personally. Give it a rating of 1 if the topic does not arouse fear in you at all. Give it a rating of 5 if the topic arouses very strong fear in you.**

| **ITEMS IN RANDOM ORDER** | Does not evoke fear at all |  |  |  | Evokes strong fear | Does not know/ does not want to answer |
| --- | --- | --- | --- | --- | --- | --- |
| People with different cultural backgrounds, migrants move to your neighbourhood | 1 | 2 | 3 | 4 | 5 | 99 |
| Somebody from your family moves abroad | 1 | 2 | 3 | 4 | 5 | 99 |
| You become vulnerable and lose control over your fate | 1 | 2 | 3 | 4 | 5 | 99 |
| The world is accelerating even faster; you are unable to catch up with the constant changes | 1 | 2 | 3 | 4 | 5 | 99 |
| You get seriously ill, hospitalized | 1 | 2 | 3 | 4 | 5 | 99 |
| Your relationship breaks up or you cannot find a partner | 1 | 2 | 3 | 4 | 5 | 99 |
| Your loved ones get hurt | 1 | 2 | 3 | 4 | 5 | 99 |
| Your financial situation becomes unstable; you will not be able to pay your bills | 1 | 2 | 3 | 4 | 5 | 99 |
| You become indebted and have to get loans | 1 | 2 | 3 | 4 | 5 | 99 |
| You lose your home, become homeless | 1 | 2 | 3 | 4 | 5 | 99 |
| You become the victim of school or workplace discrimination | 1 | 2 | 3 | 4 | 5 | 99 |
| You become a victim of crime, violent attack | 1 | 2 | 3 | 4 | 5 | 99 |

**2, Covid-19 fear index**

|  | Not at all | Rather not | Rather yes | A lot | Does not know/ does not want to answer |
| --- | --- | --- | --- | --- | --- |
| In your opinion, how serious threat does coronavirus pose to human health? | 1 | 2 | 3 | 4 | 9 |
| If you got infected with coronavirus, how serious would its consequences be? | 1 | 2 | 3 | 4 | 9 |
| Overall, to what extent do you consider coronavirus a serious threat to the health of your family? | 1 | 2 | 3 | 4 | 9 |
| How much do you fear that coronavirus infection causes serious, long-term complications even after the acute course of the illness? | 1 | 2 | 3 | 4 | 9 |

**3) Vaccinations**

|  | Yes, I would definitely vaccinate | I would probably vaccinate | I probably would not vaccinate | I certainly would not vaccinate | I have already vaccinated | Does not know/ does not want to answer |
| --- | --- | --- | --- | --- | --- | --- |
| Would you accept one of the vaccines against coronavirus? | 1 | 2 | 3 | 4 | 5 | 9 |
| If only European-American-developed vaccines were available, would you accept vaccination against coronavirus? | 1 | 2 | 3 | 4 | 5 | 9 |
| If only Chinese-developed vaccines were available, would you accept vaccination against coronavirus? | 1 | 2 | 3 | 4 | 5 | 9 |
| If only Russian-developed vaccines were available, would you accept vaccination against coronavirus? | 1 | 2 | 3 | 4 | 5 | 9 |

**4) How often do you follow the behaviours below to prevent coronavirus infection?**

| **ITEMS IN RANDOM ORDER** | Not at all | Sometimes | Mostly yes | Always | Does not know/ does not want to answer |
| --- | --- | --- | --- | --- | --- |
| If you are outside your home, you are wearing a face mask. | 1 | 2 | 3 | 4 | 99 |
| If you leave your home, whenever you have a chance to, you disinfect your hands, wash your hands with soap. | 1 | 2 | 3 | 4 | 99 |
| If you are outside your home, you will keep at least 2 metres of distance when talking to others and waiting in line. | 1 | 2 | 3 | 4 | 99 |
| Avoid public transportation as much as possible. | 1 | 2 | 3 | 4 | 99 |
| When you meet friends, acquaintances, co-workers, you avoid physical contact (handshake, hug, welcome kiss). | 1 | 2 | 3 | 4 | 99 |
| You cancel or avoid gatherings where more people would have been expected to attend. | 1 | 2 | 3 | 4 | 99 |
| You avoid contact with persons (eg.: elderly and/ or chronic patients) to whom being infected with coronavirus is particularly risky. | 1 | 2 | 3 | 4 | 99 |
| You only leave home for the most necessary reasons. | 1 | 2 | 3 | 4 | 99 |
| You take vitamins to strengthen your immune system. | 1 | 2 | 3 | 4 | 99 |

**5) In what follows, we will ask whether your human relationships have become closer, or became chilled during the period of quarantine and social distancing?**

| **ITEMS IN RANDOM ORDER** | They became much closer | They became closer | They did not change | They became more chilled | They became much more chilled | Does not have such relationship | Does not know/ does not want to answer |
| --- | --- | --- | --- | --- | --- | --- | --- |
| Family relationships | 1 | 2 | 3 | 4 | 5 | 88 | 99 |
| Romantic relationship | 1 | 2 | 3 | 4 | 5 | 88 | 99 |
| Friendships | 1 | 2 | 3 | 4 | 5 | 88 | 99 |
| Working relationships | 1 | 2 | 3 | 4 | 5 | 88 | 99 |

**6) People are worried about coronavirus and its consequences for several reasons. How worried are you about the following? Is there anything that has already happened?**

| **ITEMS IN RANDOM ORDER** | Not worried at all | Rather not worried | Rather worrying | Seriously worried | Has already happened | Does not know/ does not want to answer |
| --- | --- | --- | --- | --- | --- | --- |
| You get sick | 1 | 2 | 3 | 4 | 8 | 9 |
| Your close family members / friends get sick | 1 | 2 | 3 | 4 | 8 | 9 |
| The epidemic will reduce your income | 1 | 2 | 3 | 4 | 8 | 9 |
| You lose your job | 1 | 2 | 3 | 4 | 8 | 9 |
| You are restricted in your freedom | 1 | 2 | 3 | 4 | 8 | 9 |
| Your partner becomes nervous, aggressive during home confinement **(ONLY FOR PEOPLE LIVING IN A ROMANTIC RELATIONSHIP D3 = 1)** | 1 | 2 | 3 | 4 | 8 | 9 |

**7) Did the financial situation of your household improve or worsen since the outbreak of the coronavirus epidemic in Hungary in March 2020?**

1, Worsened

2, Did not change

3, Improved

---------------------------------------------------------------------------------------------------

99 Does not know/ does not want to answer

**8) In the following, we list statements about the COVID pandemic, please mark on a scale of 10 how much you agree with those statements!**

| **THE 5 ITEMS BELOW ARE ORDERED RANDOMLY** | Does not agree at all |  |  |  |  |  |  |  |  | Completely agree | Does not know/ does not want to answer |
| --- | --- | --- | --- | --- | --- | --- | --- | --- | --- | --- | --- |
| Coronavirus does not exist; it is made up. | 1 | 2 | 3 | 4 | 5 | 6 | 7 | 8 | 9 | 10 | 99 |
| Coronavirus was deliberately released to the world by China in order to take over the role of world leader. | 1 | 2 | 3 | 4 | 5 | 6 | 7 | 8 | 9 | 10 | 99 |
| Coronavirus was developed by pharmaceutical companies in order to help them sell their drugs and vaccines more easily. | 1 | 2 | 3 | 4 | 5 | 6 | 7 | 8 | 9 | 10 | 99 |
| COVID-19 vaccination may cause infertility; the secret goal of the vaccination is population control. | 1 | 2 | 3 | 4 | 5 | 6 | 7 | 8 | 9 | 10 | 99 |
| With the COVID-19 vaccination, a microchip may be implanted into the body secretly. | 1 | 2 | 3 | 4 | 5 | 6 | 7 | 8 | 9 | 10 | 99 |

**Declaration of data management**

(provided by our partner, Závecz Research, translated from Hungarian)

I approve of the recording of my name, address and/or telephone number, which may be used by the employees of H-Building Hungary Kft. for control of the research data. The personal data I provide will not be shared with third party, it will be deleted after the end of the project, and it will not be used in any other way.

I can withdraw this declaration at any time by sending an email to [milovecz@hreports.hu](mailto:milovecz@hreports.hu).

Acceptance of declaration:

Yes

No
